# Probe-based metagenomic pathogen detection: advancing laboratory capacity for complex diagnosis

**DOI:** 10.3389/fmicb.2025.1656831

**Published:** 2025-10-14

**Authors:** Rita Ferreira, Luís Coelho, João Dourado Santos, Daniel Sobral, Joana Isidro, Verónica Mixão, Miguel Pinto, Alexandra Nunes, Maria José Borrego, Sílvia Lopo, Mónica Oleastro, Rita Sousa, Paula Palminha, Cristina Veríssimo, Maria João Gargaté, Raquel Guiomar, Rita Cordeiro, Rita Macedo, Paula Bajanca-Lavado, Paulo Paixão, Sílvia Duarte, Luís Vieira, Vítor Borges, João Paulo Gomes

**Affiliations:** ^1^Genomics and Bioinformatics Unit, Department of Infectious Diseases, National Institute of Health Doutor Ricardo Jorge (INSA), Lisbon, Portugal; ^2^Veterinary and Animal Research Centre (CECAV), Faculty of Veterinary Medicine, Lusófona University, Lisbon, Portugal; ^3^National Reference Laboratory for Sexually Transmitted Infections, Department of Infectious Diseases, National Institute of Health Doutor Ricardo Jorge (INSA), Lisbon, Portugal; ^4^National Reference Laboratory of Gastrointestinal Infections, Department of Infectious Diseases, National Institute of Health Doutor Ricardo Jorge (INSA), Lisbon, Portugal; ^5^Centre for Vectors and Infectious Disease Research (CEVDI), Águas de Moura, Department of Infectious Diseases, National Institute of Health Doutor Ricardo Jorge (INSA), Lisbon, Portugal; ^6^National Reference Laboratory of Vaccine Preventable Diseases, Department of Infectious Diseases, National Institute of Health Doutor Ricardo Jorge (INSA), Lisbon, Portugal; ^7^National Reference Laboratory for Parasitic and Fungal Infections, Department of Infectious Diseases, National Institute of Health Doutor Ricardo Jorge (INSA), Lisbon, Portugal; ^8^National Reference Laboratory for Influenza and Other Respiratory Viruses, Department of Infectious Diseases, National Institute of Health Doutor Ricardo Jorge (INSA), Lisbon, Portugal; ^9^Emergency Response and Biopreparedness Unit, Department of Infectious Diseases, National Institute of Health Doutor Ricardo Jorge (INSA), Lisbon, Portugal; ^10^National Reference Laboratory for Tuberculosis - NRL-TB, Department of Infectious Diseases, National Institute of Health Doutor Ricardo Jorge (INSA), Lisbon, Portugal; ^11^National Reference Laboratory for Respiratory Infections by Bacterial Agents, Department of Infectious Diseases, National Institute of Health Doutor Ricardo Jorge (INSA), Lisbon, Portugal; ^12^CHRC - Nova Medical School, Universidade Nova de Lisboa, Lisbon, Portugal; ^13^Technology and Innovation Unit, Department of Human Genetics, National Institute of Health Doutor Ricardo Jorge (INSA), Lisbon, Portugal

**Keywords:** metagenomics, next-generation sequencing, mNGS, pathogen detection, INSaFLU-TELEVIR, bioinformatics analysis

## Abstract

Probe-based pathogen enrichment, followed by NGS, is a promising tool for complex diagnosis, overcoming traditional challenges of shotgun metagenomics, namely small microbial/human genetic material ratio and demanding computational resources. Here, we assessed the combined detection performance of two Illumina probe-based panels, the Respiratory and the Urinary Pathogen ID panels (RPIP and UPIP), using 99 clinical samples of 15 different matrices (e.g., cerebrospinal fluid, plasma, serum, urine, swabs, biopsies, etc.) available from Portuguese National Reference Laboratories. This sample set involved 114 “PCR-positive hits” (Ct values range of 9.7–41.3; median of 28.4) for 52 non-redundant human pathogens. For a more detailed bioinformatics assessment, as a complement of the Illumina turnkey solution (Explify), we applied an extended version of our INSaFLU-TELEVIR(+) metagenomics pipeline. Whereas Explify analyses resulted in an initial detection frequency of 73.7% (84/114), the subsequent application of INSaFLU-TELEVIR(+), including taxonomic classification followed by confirmatory read mapping, enabled an overall detection proportion of 79.8% (91/114) of the PCR-positive hits. This translated into a detection rate increment from 54.3% (19/35) to 65.7% (23/35) for bacteria, and from 85.3% (58/68) to 89.7% (61/68) for viruses. The implemented workflow was also very satisfactory for samples with qPCR Ct values above 30, with an overall detection frequency of 71.8% (28/39) when compared with the 92.0% (46/50) observed for those with Ct ≤ 30. In summary, this study validated and established a pioneering approach at the Portuguese National Institute of Health to support clinicians in complex diagnosis, contributing to advance diagnostic capabilities toward a more informed clinical decision and potential improvement of infectious disease outcomes.

## 1 Introduction

Conventional diagnosis of infectious diseases and consequent clinical treatment primarily depend on patients' signs and symptoms, laboratory biochemical parameters, imaging observations, and specific targeted laboratory tests such as culture, serological and/or molecular assays (Das et al., 2018; Lin et al., 2020; Liu et al., 2023; Zhang et al., 2023; Ramaiah et al., 2025). However, in the absence of any inklings about the etiology of disease, multiple tests are frequently required, leading to time-consuming “trial and error” procedures. While this approach provides no guarantees of the establishment of a diagnosis, it also often delays the application of a targeted treatment, with potential negative impact on patient recovery. Indeed, the undiagnosed clinical cases often lead to the prescription/administration of broad-spectrum antibiotics (Gonzales et al., 2001; Ruopp et al., 2015; Li et al., 2016), which greatly increases the risk of the emergence of multidrug-resistant bacteria, and the potential for a higher mortality rate among these mis-/un-diagnosed patients (Brown et al., 2018; Han et al., 2019). For instance, according to some literature reports, in respiratory samples, an accurate laboratory diagnosis is only reached in about 70%−80% of the cases (Ewig et al., 2002; Johansson et al., 2010), while more than half of encephalitis cases remain undiagnosed (Glaser et al., 2006; Granerod et al., 2010a; Wilson et al., 2019; Kumar, 2020; Shi et al., 2022). Even though PCR assays targeting multiple pathogen at the same time are now widely used and a syndromic diagnostic testing is frequently applied, the wide range of possible disease-causing agents at play (Granerod et al., 2010b; Wilson et al., 2014) make it infeasible to test all of them in a timely manner, contributing to the non-neglected rate of undiagnosed infections.

One approach with the potential of overcoming some of the limitations in the conventional diagnosis of infectious diseases is the complementary application of next-generation sequencing (NGS)-based metagenomics (mNGS), which consists in the sequencing of virtually all the genetic material present in a sample. Combined with the recent advances in the development of more comprehensive and high-quality reference databases and more robust bioinformatics tools (Vilsker et al., 2019; Kalantar et al., 2020; Santos et al., 2024), mNGS technology has already seen broad application to both research studies and, more recently, clinical microbiology (Besser et al., 2018; Miao et al., 2018; Ruppé and Schrenzel, 2018; Lin et al., 2023; Benoit et al., 2024).

In principle, mNGS may be applied directly to a clinical sample without any previous knowledge of suspected etiology, microbial culture or target enrichment procedure, hence enabling the potential detection of any pathogen present, including uncultivable/fastidious, unexpected or novel (Chiu, 2013; Garza and Dutilh, 2015; Charalampous et al., 2019; Chiu and Miller, 2019; Wilson et al., 2019). However, for this hypothesis-free approach to be implemented and routinely applied as a diagnostic tool in clinical practice, several challenges/limitations still need to be overcome (Greninger, 2018; Gu et al., 2019; Chiang and Dekker, 2020; Chen et al., 2022). For example, the overall cost of mNGS, *per* sample, remains fairly high when compared to routine diagnostic methods, and the often large amount of “hits” detected makes it difficult for clinicians to interpret on their own (Ruppé et al., 2017; Greninger, 2018; Zhao et al., 2024). Additionally, in absence of any pathogen enrichment or host depletion procedures, it has been shown that the great majority (>90%) of all generated sequences have host origin (Wylie et al., 2012; Hasan et al., 2016; Salzberg et al., 2016; Mongkolrattanothai et al., 2017), with a residual proportion of sequences deriving from microorganisms, which may include the actual pathogen(s), resident microbiota or even contaminant microorganisms (Simner et al., 2018). In this context, multiple efforts have been made toward the development of pathogen enrichment methodologies to boost pathogens' detection sensitivity, either by host depletion or direct pathogen capture (Cheng et al., 2022; Marquet et al., 2022; Pham et al., 2023; Chen et al., 2024; Islam Sajib et al., 2024; Yi et al., 2024). However, due to the diversity of clinical specimens (e.g., blood, stool, biopsies, etc.) and of infectious pathogenic agents (viruses, bacteria, fungi, and parasites), these targeted sequencing procedures can be more situational than true mNGS methods, requiring some clinical context for their effective application (Jacob et al., 2019). Recently, NGS methods based on the use of specific probes for the simultaneous targeted sequencing of multiple pathogens (herein referred to as targeted NGS - tNGS) have been developed, promising to strike a balance between shotgun NGS and conventional pathogen-specific detection (Gaston et al., 2022; Almas et al., 2023; Lin et al., 2023; Zhao et al., 2024). Two commercially available tNGS-based approaches, namely the Respiratory Pathogen ID/AMR Panel (RPIP) and the Urinary Pathogen ID/AMR Panel (UPIP) from Illumina, aim to rapidly and simultaneously detect a large group of human pathogens (up to 383 bacteria, viruses, fungi and parasites, collectively), enabling a broad application yet a more focused screening of the pathogens' genetic material present in clinical samples.

In the present study, we sought to assess the combined performance of the RPIP/UPIP Illumina technology to detect multiple infectious agents in a diverse set of clinical samples received by the National Institute of Health Doutor Ricardo Jorge (INSA), Portugal, toward its implementation as a complementary assay to support clinicians in complex diagnoses. For a more detailed assessment, as a complement of the Illumina turnkey bioinformatics solution (Explify, available at Illumina's BaseSpace; https://basespace.illumina.com/), we adapted our own bioinformatics metagenomic pipeline, extending the INSaFLU-TELEVIR platform (Santos et al., 2024).

## 2 Materials and methods

### 2.1 Sample selection and characterization

Samples were requested from the National Reference Laboratories (NRLs) at the Portuguese National Institute of Health (INSA) and from clinicians at external health care institutions, based on specific criteria: (*i*) to have tested positive by specific molecular methods (PCR or real-time PCR) in the course of laboratory's routine activities, for at least one of the 383 human pathogens collectively detectable by Illumina's RPIP (#20047050, M-GL-00115 v2.0 of March of 2023; https://www.illumina.com/content/dam/illumina/gcs/assembled-assets/marketing-literature/respiratory-pathogen-panel-table-m-gl-00115/respiratory-pathogen-id-amr-panel-table-m-gl-00115.pdf) and UPIP (#20090308, M-GL-01888 v1.0 as of March of 2023; https://www.illumina.com/content/dam/illumina/gcs/assembled-assets/marketing-literature/urinary-pathogen-id-amr-panel-table-m-gl-01888/urinary-pathogen-id-amr-panel-table-m-gl-01888.pdf); (*ii*) to be a clinical sample and not a cultured isolate; and (*iii*) to meet the volume requirements for downstream procedures according to sample nature (Supplementary Table 1).

Although there was no sampling date restriction, collaborating laboratories were asked to prioritize more recently collected samples in order to potentiate the integrity of the genomic material and to mirror real situations where mNGS would be requested to the NRLs for diagnosis assistance in real-time.

**Figure 1:**
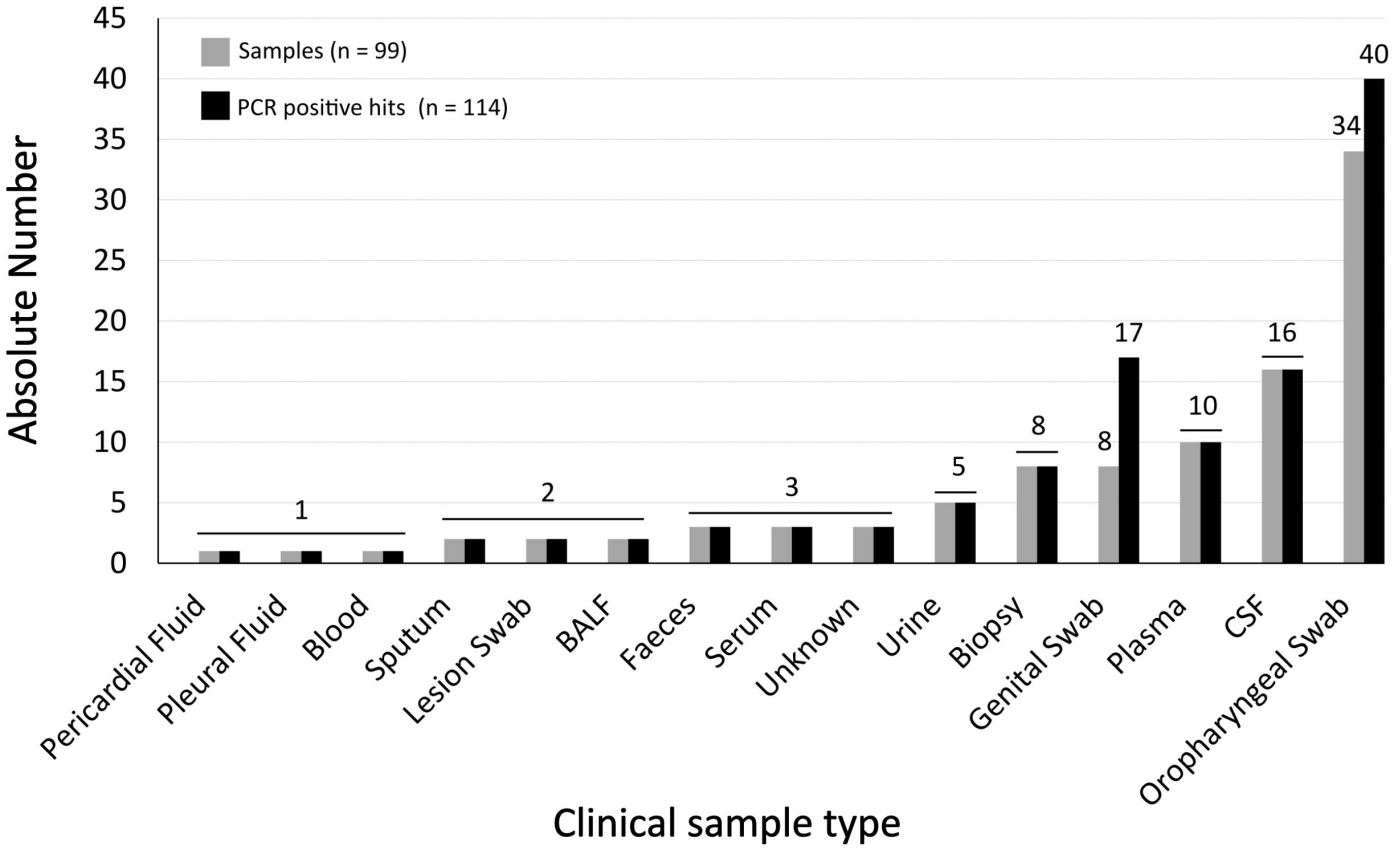
Dataset characterization. The graph shows the absolute distribution of samples (light-gray bars) and PCR positive hits (dark-gray bars) *per* sample type (horizontal axis).

**Figure 2:**
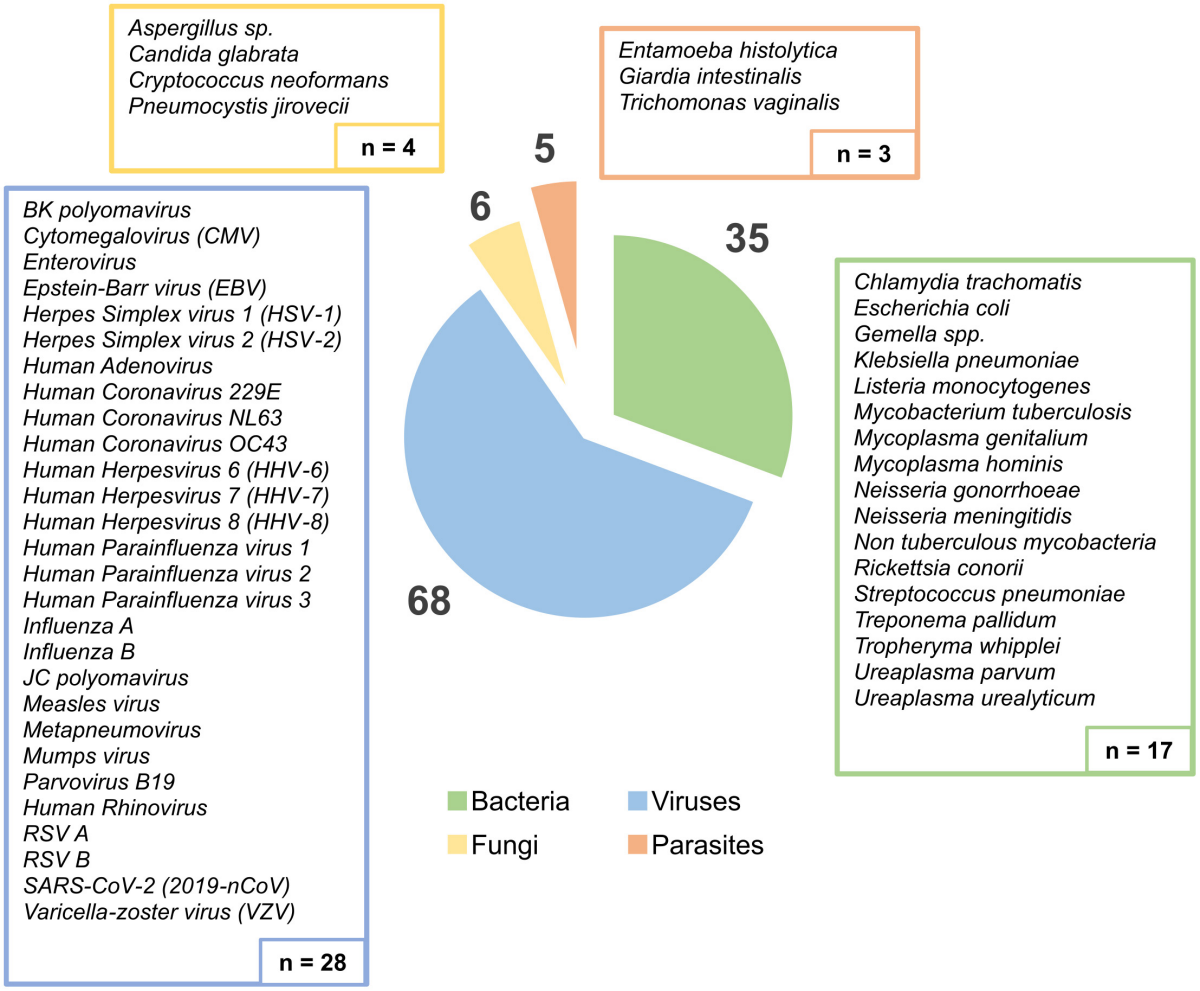
Distribution of all PCR-identified pathogens present in the samples, according to pathogen group (Bacteria, Viruses, Fungi, and Parasites). The boxes show the list and absolute number of different human pathogens detected by PCR assays (*n* = 52 non-redundant pathogens), while the circular graph displays the distribution of the total PCR positive hits (*n* = 114).

Overall, for the assessment of the tNGS performance, a total of 99 samples were collected, of which 85 were provided by the NRLs, while 14 were provided by clinicians from external health care institutions. Samples originated from different biological matrices, namely biopsy, blood, bronchoalveolar lavage fluid (BALF), cerebrospinal fluid (CSF), feces, genital swab, lesion swab, oropharyngeal swab, pericardial fluid, plasma, pleural fluid, serum, sputum, and urine (Figure 1). This sample set enrolled a total of 114 “PCR positive hits” (68 viruses, 35 bacteria, 6 fungi, and 5 parasites) previously identified by (q)PCR by the participating laboratories, covering a total of 52 non-redundant human pathogens (Figure 2). Indeed, presence of more than one pathogen was reported in some genital (*n* = 4) and oropharyngeal (*n* = 6) swabs, totalizing 17 pathogens in 8 genital samples and 40 pathogens in 34 oropharyngeal samples. Of the 114 PCR positive hits, 23 had been identified by conventional PCR, while 91 were diagnosed through specific qPCR testing, thus real-time PCR cycle threshold (Ct) values were available, and ranged from 9.7 to a maximum of 41.3 (median of 28.4).

### 2.2 Nucleic acid extraction

Samples (*n* = 99) enrolled in this study underwent two different extraction procedures: (*i*) 69 of the samples provided by INSA's Reference Laboratories followed the laboratories' respective routine diagnosis procedures of sample treatment and extraction using the automated extraction system EMAG^®^ (BioMerieux, Portugal); (*ii*) 30 samples, including 14 provided by the external health care institutions, were subjected to the extraction procedure referred to in the *Explify Respiratory Pathogen ID/AMR Panel User Guide: Sample Preparation and Sequencing Library Preparation* (CUS.USRG.9001.03). The latter allowed for the independent DNA and RNA extraction by using the Quick-DNA/RNA MiniPrep Plus kit (version 2.1.6, Zymo Research), taking into consideration each sample's nature in order to carry out the appropriate pre-extraction sample preparation procedure, according to manufacturers' instructions (Supplementary Table 1). Briefly, the material collected by the Spin-Away Filter proceeded to DNA extraction, and the flow-through proceeded to RNA extraction using the ZymoSpin™ IIICG Columns. DNase I treatment was also included in all RNA extractions, according to manufacturer's instructions. Finally, both the DNAs and the RNAs were eluted in DNase/RNase-Free Water. Contrarily to the 69 samples that were retrospectively selected from the sample collection of each NRL, for the remaining 30 samples the extraction session included one negative and one positive controls (NC and PC, respectively) to be sequenced alongside the samples. All NC were composed of sterile DPBS (Gibco) in equal starting volumes as the samples of that particular nucleic acids extraction session. A ZymoBIOMICS community II sample (Zymo Research, CA, USA) was processed as the PC *per* extraction session, and prepared according to manufacturer's instructions. Finally, and because Quick-DNA/RNA MiniPrep Plus kit (Zymo Research, CA, USA) allows for the independent extraction of DNA and RNA, two different internal controls (IC), the T7 (DNA) and the MS2 (RNA) bacteriophages (T7-UP-LEX and MS2-UP-LEX, respectively, from Microbiologics, MN, USA), were added to every sample, including all NCs and PCs, at the beginning of each extraction session. The detection of the spiked-in T7 and MS2 aimed to confirm the success of DNA and RNA extraction procedures.

### 2.3 Library preparation and enrichment

RPIP and/or UPIP library preparation was conducted for all samples, depending on the presence of the pathogen (previously detected by PCR) in the list of organisms covered by each panel. Whenever a pathogen was covered by the two panels, the respective samples were subjected to both RPIP and UPIP procedures, not only to compare their individual performance, but also to assess the benefits of their simultaneous application.

#### 2.3.1 RPIP library preparation and enrichment

Sequencing libraries were prepared according to Illumina/IDbyDNA Respiratory Pathogen ID/AMR Panel (RPIP) protocol (Explify, CUS.USRG.9001.03). Briefly, cDNA synthesized from extracted RNA was combined with extracted DNA in equal volumes. Libraries were constructed by tagmentation and adapter ligation with IDT for Illumina DNA/RNA UD Indexes (Illumina, Inc) followed by amplification for 18 cycles. Indexed libraries were pooled in triplicate by volume and hybridized with the Respiratory Pathogen ID/AMR Oligo Panel, leaving to stand at 58 °C, for approximately 20 h. The captured libraries were amplified for 17 cycles, followed by a purification step using AMPure XP Beads (Beckman Coulter).

#### 2.3.2 UPIP library preparation and enrichment

Sequencing libraries for Illumina Urinary Pathogen ID/AMR Panel (UPIP) (Illumina, Inc) were constructed following the Library Prep Instrument workflow described in the Illumina DNA Prep with Enrichment Reference Guide (document #1000000048041), considering the deviations described in UPIP Library Prep Protocol Deviations (document #200028760). Shortly, extracted DNA was tagmented and indexed with IDT for Illumina DNA/RNA UD Indexes (Illumina, Inc). Libraries were amplified for 18 cycles and cleaned with AMPure XP Beads (Beckman Coulter). The indexed libraries were pooled in triplicate by volume for hybridization with the Urinary Pathogen ID/AMR Enrichment Probes, leaving to stand at 58 °C, for approximately 20 h. The captured libraries were amplified for 17 cycles, followed by a purification step using AMPure XP Beads (Beckman Coulter).

#### 2.3.3 Sequencing

Quality analysis of pooled enriched libraries was performed with the Fragment Analyser system and the HS NGS Fragment (1–6,000bp) kit (Agilent Technologies, Austin, TX, USA) and subsequently normalized to 4 nM. A final pool was done with equal volumes of all enriched library pools and quantified using a Qubit 3.0 fluorometer with Qubit™ dsDNA HS Assay kit (Invitrogen, Waltham, MA, USA). For samples run on MiSeq and NextSeq 550, denaturation and dilution of the final pool was conducted in accordance to the “Denature and Dilute Libraries Guide” of each platform, as so the respective loading concentrations. For the NextSeq 2000, libraries were denatured on-board using the loading concentration indicated in the “NextSeq 1000 and 2000 Sequencing System Guide” (Document # 1000000109376 v05, Illumina, August 2021). Sequencing was performed with 150 bp single-end read length, with the run design targeting an expected read yield of at least 1 M reads per sample in a single-end run.

### 2.4 Bioinformatics analysis

**Figure 3:**
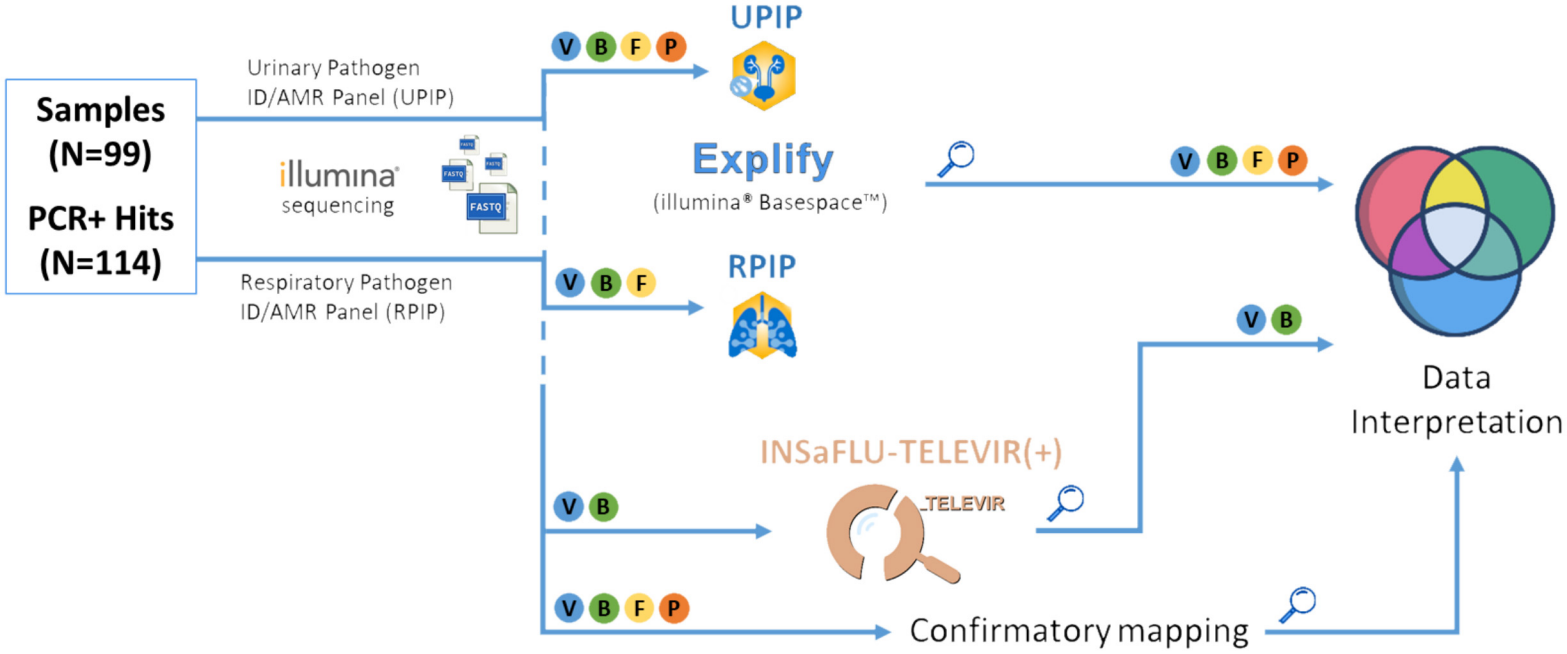
Bioinformatic pipeline for pathogen identification. Illustrative representation of the bioinformatics steps of the tNGS pipeline used in this study. “Magnifying glass” symbol: key checkpoints for assessment of pathogen detection rates, i.e., after analysis by Explify (only), INSaFLU-TELEVIR(+) taxonomic classifiers (only), and combination, followed by confirmatory mapping. V, Viruses; B, Bacteria; F, Fungi; P, Parasites.

We conducted a multi-step strategy (illustrated in Figure 3) to evaluate the performance of the probe-based RPIP/UPIP Illumina technology and associated bioinformatics pipeline—both individual steps and the workflow as a whole—toward implementation and routine adoption at INSA. First, we verified the presence of the expected pathogen (PCR positive hits) within the results generated by Explify (Illumina BaseSpace) (2.4.1. Step A) and the taxonomic classifiers incorporated in the extended INSaFLU-TELEVIR metagenomics workflow [TELEVIR(+)] (2.4.2. Step B). Finally, direct read mapping (2.4.3. Step C) against representative reference sequences of the expected pathogens was carried out as a final validation step. This step served to confirm PCR results as true hits at the bioinformatics level, while refining the interpretation and assessment of detection performance across intermediate steps [Explify and INSaFLU-TELEVIR(+) taxonomic classification]. Details for each step of the analysis are presented below.

#### 2.4.1 Step A: Explify bioinformatics analysis

Sequencing data resultant from RPIP and UPIP sample sets were analyzed using the Explify RPIP (v2.1.1, IDbyDNA) and the Explify UPIP (v1.1.0, IDbyDNA) data analysis applications, respectively, available at the Illumina BaseSpace Sequencing Hub (https://basespace.illumina.com/). The Explify RPIP and UPIP apps were launched with the following options selected: (*i*) Read Quality Control (QC) (low quality read trimming and filtering); (*ii*) default reporting of all microorganisms; and (*iii*) quantitative analysis of internal control was set as “NONE.” Considering that some of the expected pathogens were known to be present in a very low load (>30 Ct values, as determined by specific qPCR), all expected pathogens present in Explify's panels' reports showing ≥ 1reads per kilobase per million mapped reads (RPKM) were accounted for as positive detections by this bioinformatics method, regardless of other quality parameters also reported, such as probe coverage or median depth. As an additional quality control check, we confirmed that all PC and IC were successfully detected by Explify applications in all samples that were subjected to spike-in during nucleic acid extraction, leading to no post-NGS sample exclusion. Regarding NC, we noticed that three microorganisms were recurrently detected (*Pseudomonas putida, Stenotrophomonas maltophilia*, and *Delftia acidovorans*), suggesting potential contamination from the reagents used or introduced from the laboratory environment. Curiously, these same contaminants have been previously described elsewhere as common contaminants (Laurence et al., 2014; Salter et al., 2014; Jagare et al., 2023).

#### 2.4.2 Step B: INSaFLU-TELEVIR(+) bioinformatics analysis

INSaFLU-TELEVIR is a free and user-oriented web-based platform for robust and timely viral pathogen identification and genomic surveillance (Borges et al., 2018; Santos et al., 2024). The metagenomics module TELEVIR (Santos et al., 2024) allows running complex workflows for viral detection, covering several combinations of steps (e.g., with/without viral enrichment or host depletion), classification software [e.g., Kaiju (Menzel et al., 2016), Kraken2 (Wood et al., 2019), Centrifuge (Kim et al., 2016), FastViromeExplorer (Tithi et al., 2018)] and databases, and produces mapping reports that leverage the combined output of multiple software (either in pairs of contigs/reads classifiers in the case of a single workflow or across workflows).

In this study, in order to enhance the applicability of the existing platform to the conducted multi-pathogen tNGS evaluation, we extended our local installation of the INSaFLU-TELEVIR platform (https://github.com/INSaFLU/docker) to include the bacterial subset of the NCBI Refseq database, as well as two indices of the same, built using the Centrifuge and Kraken2 algorithms (Kim et al., 2016; Wood et al., 2019) (the Centrifuge bacterial index was built on November 22nd 2023; the Kraken2 bacterial index was downloaded on July 13th 2023; the bacterial subset of the RefSeq nucleotide database was downloaded on November 20th 2023). We will refer to this extended local version (Virus and Bacteria) of the TELEVIR module as TELEVIR(+) from here on out. For viruses, the applied workflow is also available through the free online tool INSaFLU-TELEVIR v.2.1.0 (https://insaflu.insa.pt/).

In summary, TELEVIR projects were created, each containing all samples, in order to deploy bacterial and viral metagenomics pipelines separately (see Supplementary File for details). Two bioinformatics classifiers, for which both viral and bacterial indices were available, Kraken2 and Centrifuge, were used to classify reads. All samples were subject to pre-processing, enrichment and host depletion (*in silico*) as part of the TELEVIR(+) workflows deployed. Due to resource and time constraints, by design, TELEVIR(+) first sorts classifier “hits” from a given workflow and then selects only the top for automatic confirmatory mapping. This threshold, a user-defined setting, implies that hits not automatically selected for confirmatory mapping are more likely to go unnoticed. In the present study, for the purposes of establishing a realistic criterion of identification, we considered only as “TELEVIR classifier positive hits” those that obtained a ranking of 20 or less after combined read/contig sorting. The selection of this threshold relied on previous observations that the proportion of true-positive hits selected for mapping reached a plateau at around 15, with minor detection gains above that threshold (Santos et al., 2024).

Following TELEVIR(+) metagenomics analysis, “True positives” were determined by matching classifier report hits (which return taxonomic identifiers) to the descriptions of pathogens verified to be present in each sample by the respective PCR/qPCR tests (see Supplementary File). Of note, samples of fungal and parasitic targets were removed from all analysis involving direct descriptions or comparisons between Explify and INSaFLU-TELEVIR(+) results, as the latter was not yet designed to analyse metagenomics data of organisms other than viruses or bacteria.

#### 2.4.3 Step C: Confirmatory mapping

We established as *bioinformatics gold standard* the result of a direct mapping of post-QC read sequencing data against the representative genome sequences of the expected pathogens (PCR-positive hits) in the respective samples (see Supplementary File). A pathogenic target was deemed suitable for evaluation if at least one non-spurious alignment is achieved through direct mapping [and after visual inspection with IGV (Robinson et al., 2011)]. Of note, confirmatory mapping was conducted via INSaFLU-TELEVIR(+) for viruses and bacteria. For fungal and parasitic hits, this step was conducted outside the platform, using the same programs and parameters (Supplementary File).

### 2.5 Cycle threshold and FNR analysis

Because of the clinical framework of the current study data, it is not possible for us to report on pathogens other than those identified by medical request. As such, it is not possible to study False Positives or even other True Positives, and ultimately to extract estimates of precision. In this context, we limited the analysis to False Negative Ratios (FNR). FNR were analyzed within the framework of two different tests of the presence of pathogens within a sample:

i. A test relying on identification by Explify panel only.
ii. A test relying on the identification by either Explify panel or the presence of that pathogen in any TELEVIR(+) classifier report with a rank not higher than 20.

In each case, the subsequent analysis of the relation between qPCR cycle thresholds (Cts) and the FNR followed the same procedure.

We set out to determine the likely proportion of false negative observations given known PCR Cts. Given the diverse but limited size of our dataset, this insight was studied across pathogens and sample types and not within specific categories. To do this, we first separated “True Positive” and “False Negative” observations and extracted a kernel density estimate of the likelihood of Cts across the global range for each category. Kernel bandwidth is extracted from the data using a 0.2 quantile (Pedregosa et al., 2011). Finally, following a bayesian framework, assuming an uniform prior distribution, at each step in our range of Cts, we established the likelihood of a False Negative given the Ct value by dividing the Likelihood of False Negative Cts, pondered by the total probability of a False Negative (i.e., multiplied by the proportion of False Negatives across the dataset), by the likelihood of that Ct (calculated as the sum of the Likelihood of False Negative and True Positive Cts).

Bootstrap estimates used to estimate variance and to fit second degree polynomial models were performed on the result of applying the above procedure on bootstrap-resample datasets of the original data. Data was binned at each step into non-overlapping, consecutive bins of 3 Cts spanning the range of available data. Polynomial model fitting was performed using binned data.

## 3 Results

### 3.1 tNGS Illumina's RPIP and UPIP pathogen detection using Explify

**Figure 4:**
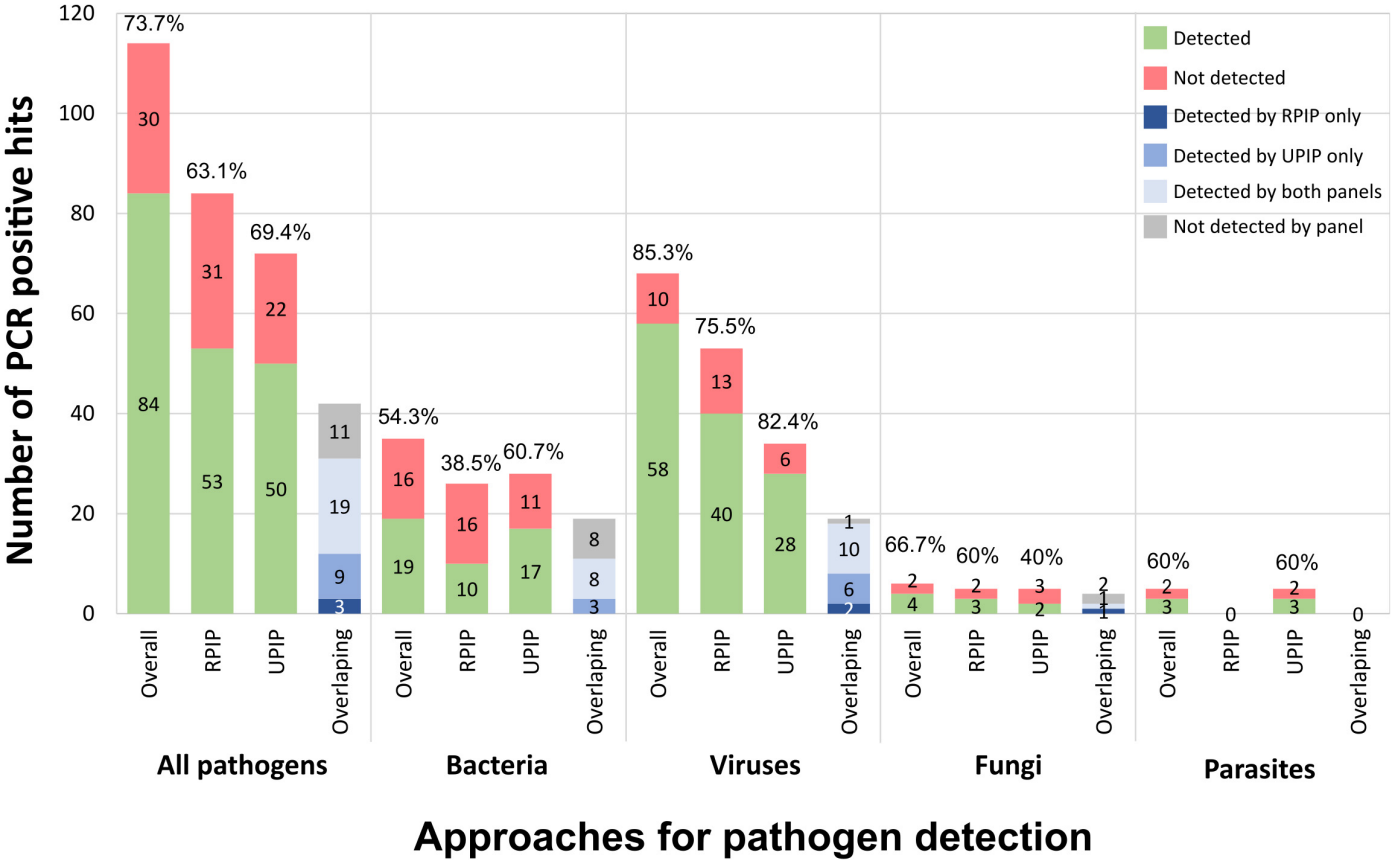
Explify pathogen detection using Illumina RPIP and UPIP panels. The graphs shows the absolute number of PCR positive hits detected (in green) and not detected (in red) by Explify according to pathogens' group, when considering (from left to right): the combined results (both panels), only the RPIP, and only the UPIP. The relative detection frequency is indicated above each bar. In each group, the right bar represents the detection distribution (per panel or combined) for the 42 pathogens covered by both panels (dark blue: only detected by RPIP; intermediate blue: only detected by UPIP; light blue: detected by both panels; gray: not detected by either panel).

The present study enrolled 99 clinical samples from 15 different sample types, involving a total of 114 “PCR positive hits” (Figure 1). To determine the performance of the Illumina RPIP and UPIP panels, tNGS pathogen detection frequency was independently assessed for each panel using the respective Explify application for data analysis. Each Illumina panel targets different groups of pathogens (although partially overlapping), with 42, 30, and 42 out of 114 PCR positive hits from our dataset being covered only by RPIP, UPIP, or both panels, respectively (Supplementary Data). Explify analyses resulted in a global detection frequency of 63.1% (53/84) for RPIP and 69.4% (50/72) for UPIP (Figure 4). Viruses were the most efficiently detected group of pathogens, with detection frequencies of 75.5% (40/53) and 82.4% (28/34) by RPIP and UPIP, respectively (Figure 4). The main difference between panels was observed for bacteria, in which RPIP performed more poorly (38.5%; 10/26) when compared to the UPIP (60.7%; 17/28) (Figure 4). Due to the limited representation of fungi and parasites in our dataset, detection frequencies for individual panels were not informative (Figure 4), being only valued when data from both panels were combined (see below).

When strictly looking at pathogens that are simultaneously covered by RPIP and UPIP (*n* = 42), UPIP again yielded better results, detecting 66.7% (28/42) of the hits when compared with 52.4% (22/42) achieved by RPIP. Still, the combination of both panels allowed an overall Explify detection of 73.8% (31/42) of the PCR-positive hits in this subset of pathogens (Figure 4; Supplementary Data). For example, for viruses (*n* = 19), while RPIP and UPIP detected 63.2% (12/19) and 84.2% (16/19), respectively, the joint use rendered a 94.7% (18/19) detection frequency.

In this context, we assessed the tNGS performance when merging the Explify results of UPIP/RPIP panels for the whole dataset. The combination of both panels yielded an overall detection frequency of 73.7% (84/114). By taxonomic group, the frequency values were 85.3% (58/68) for viruses, 54.3% (19/35) for bacteria, 66.7% for fungi and 60% for parasites (Figure 4; Supplementary Data).

### 3.2 tNGS Illumina's RPIP and UPIP pathogen detection using the combination of Explify and INSaFLU-TELEVIR(+) taxonomic classifiers

To confirm and complement the Explify analyses, we used our in-house metagenomics analysis pipeline TELEVIR(+) (Figure 3, and methods). As the TELEVIR(+) classification step only includes viruses and bacteria, samples of fungal and parasitic targets were excluded from this step (Figure 3). In total, 103 PCR-positive hits (35 bacterial and 68 viral), accounting for >90% targets on our sample collection, were considered in this combined assessment of Explify and TELEVIR(+) classification performances.

**Figure 5:**
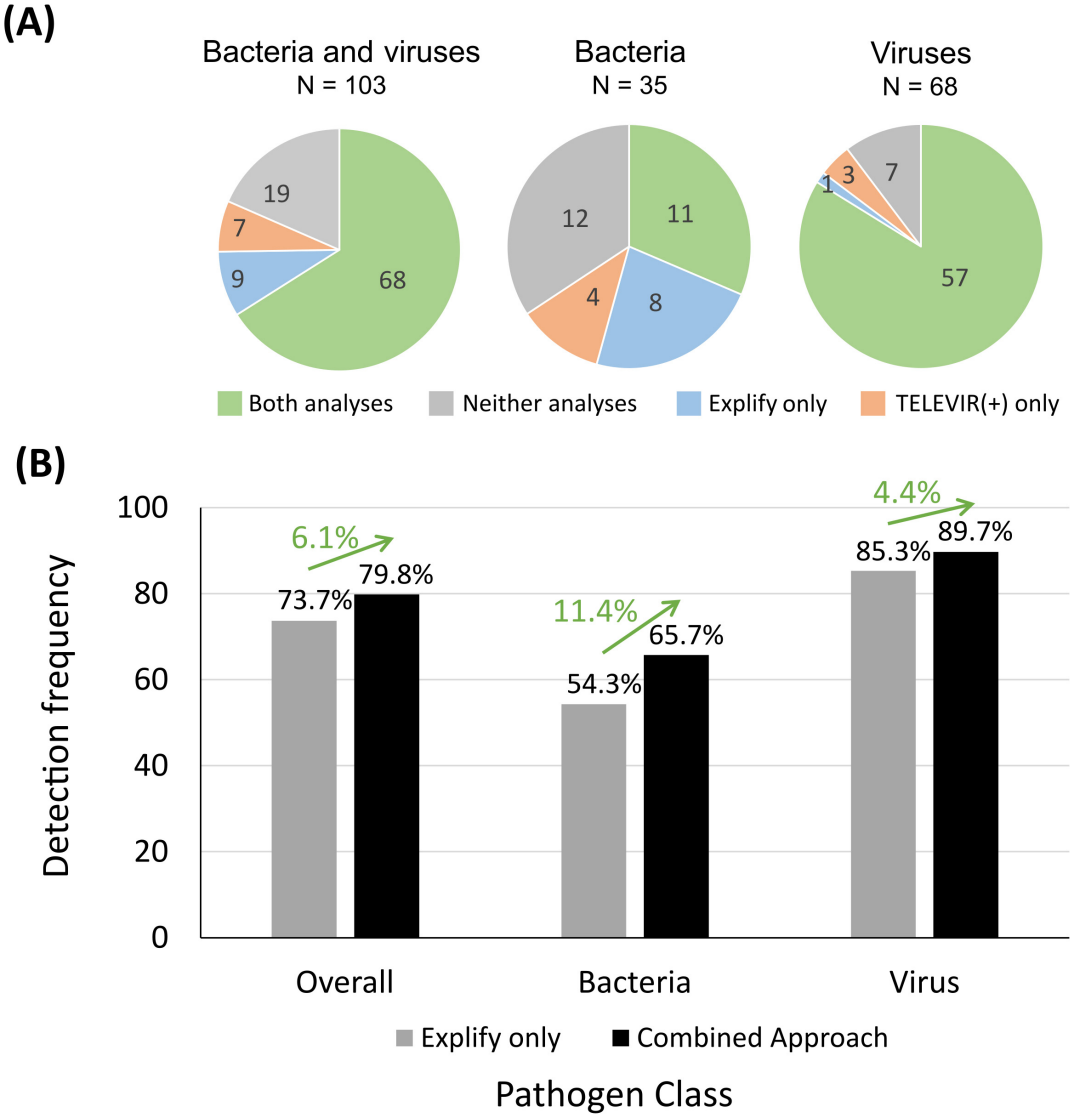
Number of bacterial and viral PCR positive hits detected by using Explify and TELEVIR(+) classification tools and detection frequency determined. In **(A)**, each pie chart represents a group of pathogens: “Bacteria and viruses” (left), “Bacteria” only (middle) and “Viruses” only (right). Pie sections indicate the relative number (their dimension) and the respective absolute number (indicated within each section) of PCR positive hits detected by both analyses (green), only by the Explify (blue), only by the TELEVIR(+) classification step (orange) and by neither (gray). The graph in **(B)** shows the detection frequencies (vertical axis) considering all PCR positive hits (“Overall”; *n* = 114), as well as within the bacterial (*n* = 35) and in the viral (*n* = 68) groups (horizontal axis), when using the Explify (only) approach (gray bars) and when using the combined approach (black bars). Each frequency value is indicated above the respective bar and the impact on estimates in using the combined approach is represented as a green arrow with the respective proportion gain above each one of them.

In this subset, Explify detected 74.8% (77/103) of the PCR positive hits (Figure 5A). Meanwhile, the TELEVIR(+) classification step—limited to the top 20 hits of any classifier (threshold for automatic hit selection for confirmatory mapping)—reported 72.8% (75/103) of the expected hits. These included 68 out of the 77 hits detected by Explify and seven newly detected hits *Ureaplasma urealyticum* (*n* = 1), *Gemella spp*. (*n* = 2), *Neisseria meningitidis* (*n* = 1), *Human Herpesvirus 6* (HHV-6) (*n* = 1), *Human Herpesvirus 8* (HHV-8) (*n* = 1), and *Enterovirus* (*n* = 1) (Figure 5A). Of note, *U. urealyticum, N. meningitides*, and HHV-6 could have been detected by either panel (RPIP and UPIP), however these pathogens were only detected by TELEVIR(+) classifiers. In total, the combination of Explify and INSaFLU-TELEVIR(+) taxonomic classifiers resulted in a combined detection frequency of 81.6% (84/103). When comparing with “Explify only” results (previous section) by group, the combined pathogen detection resulted in an increment of detection frequency, from 54.3% to 65.7% (23/35) for the bacteria, and from 85.3% to 89.7% (61/68) for the viruses (Figure 5B; Supplementary Table 2). Furthermore, no major differences were observed between Gram-positive/Gram-negative bacteria nor DNA/RNA viruses (Supplementary Data; Supplementary File).

### 3.3 Confirmatory mapping

All PCR positive bacterial and viral hits identified by combining Explify and TELEVIR(+) classification (84/103), as well as the PCR positive fungal and parasite hits identified by Explify (7/11), were fully corroborated via confirmatory mapping of reads against known references of the pathogenic taxa (Supplementary Data). This combined analysis led to an overall detection frequency of the implemented tNGS workflow of 79.8% (91/114) (Figure 5B; Supplementary Table 2). The 23 out of 114 PCR-positive hits that failed using this workflow fall within two categories: (*i*) 13 PCR positive hits (five viruses, four bacteria, two fungi, and two parasites) could not be confirmed by mapping, meaning that most likely no reads were generated during Illumina RPIP/UPIP sequencing; and (*ii*) 10 PCR positive hits that could be confirmed by mapping (two viruses and eight bacteria; detailed in Supplementary Data). A fine inspection of the latter showed that they were identified by TELEVIR(+) classifiers ranking between 43 and 589, thus not meeting the criteria for identification assumed for this intermediate workflow step (i.e., their ranking positions were all below the top 20 hits).

### 3.4 Detection performance of the combined tNGS workflow according to qPCR cycle threshold

**Figure 6:**
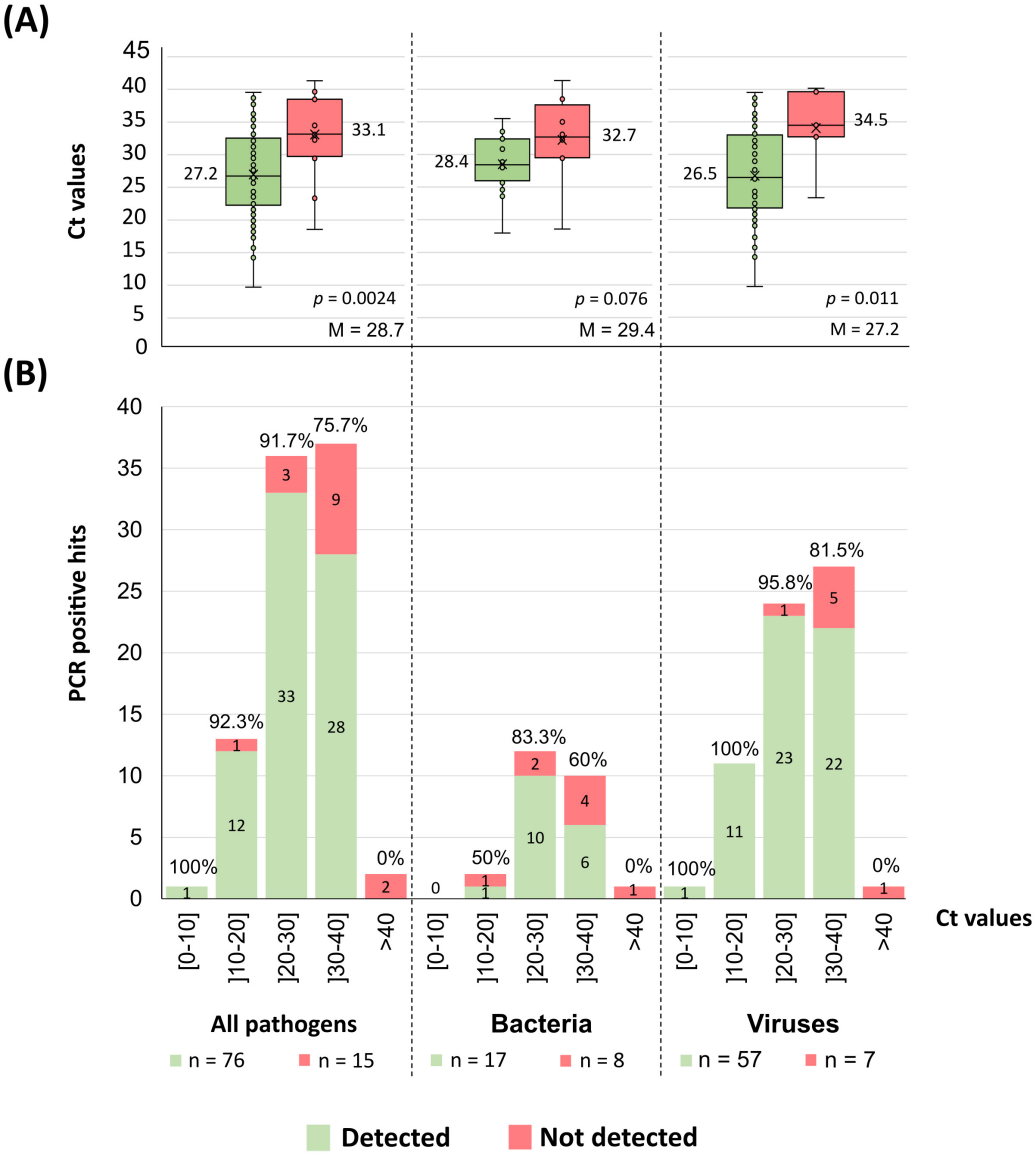
Pathogens tNGS detection according to qPCR Ct values. **(A)** The graph shows the dispersion of the pathogens Ct values (as in boxplots) according to the detection result obtained with the implemented mNGS workflow (Figure 3). Alongside each boxplot is shown the median Ct value of the respective data set, as well as the overall Ct median value (“M”) determined within each group of pathogens. Each figure also displays the *p*-value of a Mann-Whitney U-test comparing the means of both groups. Of note, the “All pathogens” aggregated plots include all PCR positive hits with available Ct values (qPCR values were not available for fungal hits and only two values were available for parasites, hindering any attempts to correlate detection for these two groups individually). **(B)** The graph shows the number of PCR positive hits, sorted by the Ct value shown as discrete intervals. The absolute number of detected (green) and non-detected (red) PCR positive hits are indicated within the respective section of each bar. Also shown is the detection frequency above the respective bar.

For 91 of the 114 PCR positive hits in our dataset, qPCR Ct values were available and ranged from 9.7 to 41.3 (median = 28.4) (Supplementary Data). As expected, the PCR positive hits that were detected by the implemented workflow tended to present lower Ct values (proxy of higher pathogen load) than those that were not detected (*p* = 0.0024; Figure 6A). Overall, the large majority (92.0%−46/50) of the PCR positive hits with Ct ≤ 30 was detected by the implemented tNGS workflow, with the detection frequency decreasing to 71.8% (28/39) for PCR positive hits with Ct values above 30 (Figure 6B). Noticeably, of the 13 PCR positive hits that could not be confirmed by mapping (see previous section), six had Ct values available being all above 32.5 (Supplementary Data).

**Figure 7:**
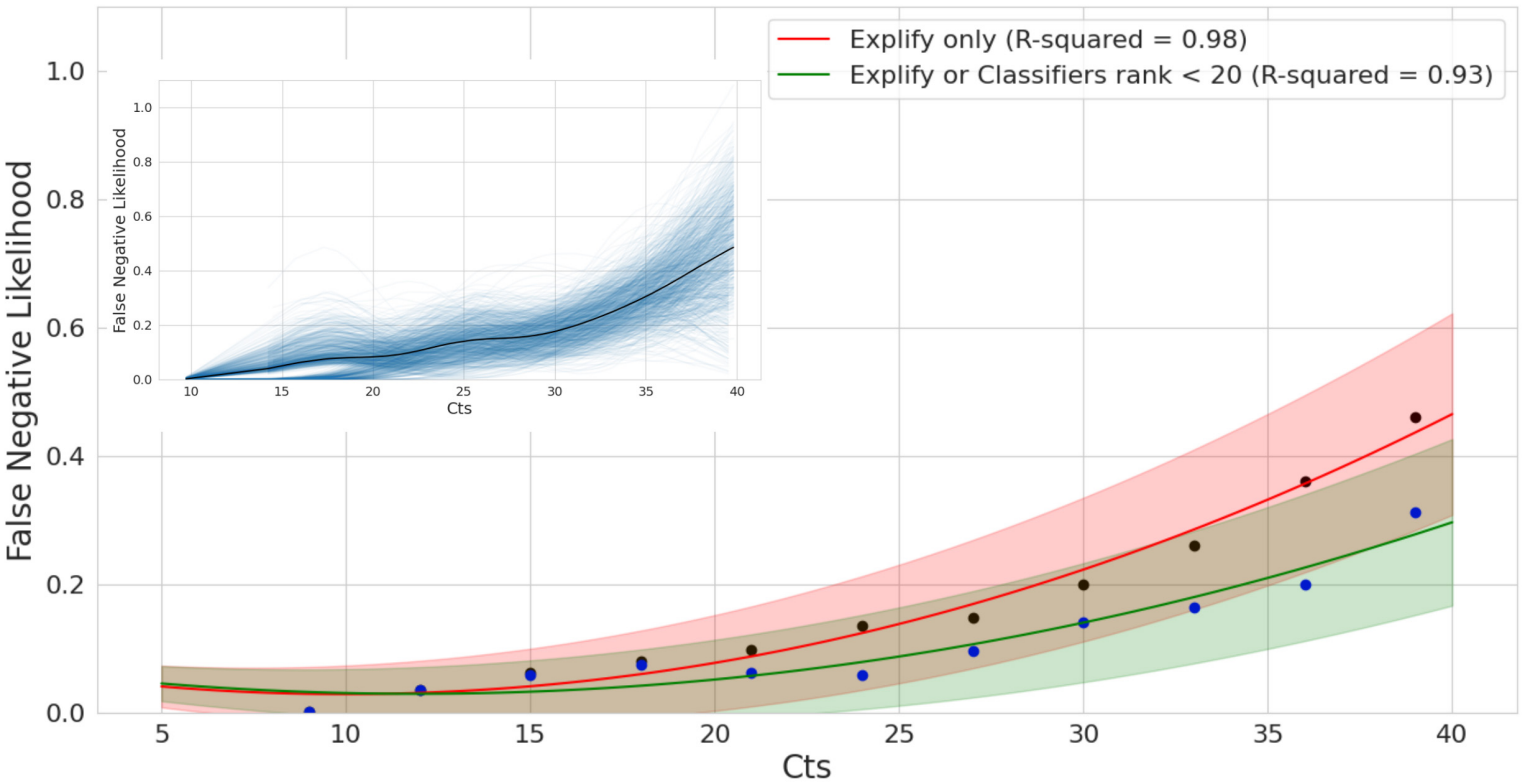
False Negative Likelihood of mNGS as a function of qPCR Ct. Bootstrap analysis and model fitting of False Negative Ratios for exact tests using Explify only (red) and Explify in combination with TELEVIR(+). Results combined for bacteria and viral pathogens. Solid lines represent model estimates following bootstrap analysis, with lower and upper bounds at the 90% confidence intervals following correction for sampling error. Black and blue dots correspond to Explify only and combined Explify and TELEVIR(+) binned averages, respectively.

To more systematically evaluate the impact of Ct on mNGS detection performance, we performed a bootstrap analysis of the relationship between tNGS detection failure (false negative ratio) and qPCR Ct values (Figure 7). This analysis revealed a strongly supported upward trend, with the likelihood of false negatives increasing with qPCR Ct values. When Explify is combined with TELEVIR(+) the slope of this trend is significantly reduced. For example, for Ct values as high as 35, Explify alone presents a likelihood of a false negative result of 33% (±13%), a value that decreases to 21% (±11%) when complemented with TELEVIR(+). Overall, our analysis suggests that, by using the implemented, combined tNGS workflow, a false negative rate of 9% (±8%) and 14% (±9%) can be expected at Cts of 25 and 30, respectively.

## 4 Discussion

Clinical metagenomics sequencing is nowadays recognized as a powerful tool for pathogen detection, mainly to support difficult diagnosis (Chiu and Miller, 2019; Chen et al., 2020). In this regard, the NRLs at the Portuguese National Institute of Health are being increasingly requested to support hospitals in resolving challenging clinical cases through mNGS, mostly when traditional diagnostic assays fail to identify the etiologic agent of the presumable infections. Due to the high diversity of clinical specimens (e.g., blood, CSF, biopsies, etc.) and infectious agents (from viruses and bacteria to fungi and parasites) that INSA has to be prepared to handle, this study aimed to assess the performance of the probe-based RPIP/UPIP Illumina technology (both panels jointly cover a total of 383 targets, 73 overlapping), and associated bioinformatics, toward its implementation and employment at INSA. Indeed, although some studies have already evaluated the performance of probe-based tNGS methods for pathogen detection (Child et al., 2023; Buddle et al., 2024; Mourik et al., 2024), there is still very limited information on it, in particular regarding the Illumina RPIP and UPIP panels, in which, to our knowledge, only one panel was tested per study on scarce clinical sample types (BALF or urine) (Gaston et al., 2022; Almas et al., 2023; Lin et al., 2023). The present study was designed to reflect the anticipated high heterogeneity of clinical samples and pathogens, simulating the real-world scenario in which requests for metagenomics diagnostics arise from diverse clinical situations. As such, our study included 99 samples, of 15 different sample types, encompassing 114 PCR-positive hits, of 52 non-redundant pathogenic taxa, covering mostly virus and bacteria, but also fungi and parasites. Several samples represented co-detections, and pathogens were present at a wide range of concentrations (measured through qPCR Ct values). Taking the PCR-positive hits as reference, we first observed that the combination of both panels, rather than their separate use, potentiated the detection of pathogens covered by both probe panels (Figure 4). As such, the downstream evaluation of the performance of the implemented workflow, step-by-step and as a whole, relied on the combined results. The first assessment consisted on evaluating the results of the commercial turnkey bioinformatics solution provided by Illumina (Explify), which enabled the detection of the majority (73.7%) of the PCR-positive hits (Figure 4). The subsequent application of advanced bioinformatics, including an intermediate step of taxonomic classification and a final step of confirmatory mapping through INSaFLU-TELEVIR(+) (Figure 3), allowed us not only to recover seven PCR positive hits not detected by Explify, but also to gather robust bioinformatics evidence of pathogen detection (through the confirmation of pathogen-specific reads by mapping against representative reference genomes). Globally, the application of the whole implemented workflow rendered an overall detection proportion of 79.8% (91 out of 114) of the PCR-positive hits (Figure 5; Supplementary Table 2). Still, this value could have been improved by considering the 10 hits that yielded reads but that did not meet the adopted criteria for automatic mapping, i.e., being among the top 20 hits after the intermediate workflow step of taxonomic classification. While this scenario unveiled a potential limitation of the implemented workflow, the dispersed TELEVIR(+) rankings (43rd−589th) and Ct values (23–40) suggest that identifying such “needles in a haystack” involves balancing the need for a rapid clinical response with the time required for ultra-deep computational analysis and confirmation of a large number of hits. In this regard, the developed and implemented TELEVIR(+) (an open-sourced tool) offers multiple functionalities (from hypothesis-free pathogen detection to target screening) that allow users to tailor the metagenomics investigation effort to the context of the clinical request.

We also observed different performances across the main pathogen groups. The implemented workflow with advanced bioinformatics yielded detection improvements of 11.4% for bacteria and 4.4% for viruses compared to the Explify-only bioinformatics, yielding final detection proportions of 65.7% (23/35) for bacteria and, noticeably, of 89.7% (61/68) for viruses (Figure 5). In general, these detection rates align well with clinical mNGS results reported in the literature, with several (prospective or retrospective) studies showing that mNGS methods provided an added-value to clinical diagnosis, while revealing that its accuracy may depend on the pathogen, type of infection/sample analyzed, treatment administration prior to sample collection and sequencing technology used (Blauwkamp et al., 2019; Miller et al., 2019; Chen et al., 2020; Deng et al., 2022; Fu et al., 2022; Kalantar et al., 2022). In our study, the heterogeneous performance cannot be disconnected from the sample type and the enrolled pathogen. For instance, whereas we detected 5/5 of the PCR-positive hits of HSV-1 (present in four different sample types, with Ct values between 23 and 40), none of the three *Chlamydia trachomatis* positive samples were detected by the implemented workflow. As the qPCR Ct values for the latter ranged from 18.57–33.08 (suggestive of reasonable bacterial load range), we cannot discard a scenario where consistent false negative hits for some pathogens are due to poor target probe design or quantity. Still, a more robust analysis, including a high number of samples per sample type as well as a higher amount of positive hits per pathogen, would be required to reach more definitive conclusions on this topic.

Not unexpectedly, the tNGS implemented workflow presented a significantly (*p* = 0.0024) higher detection performance for samples with lower qPCR Ct values (proxy of higher pathogen load). Still, the results were also very satisfactory for samples with Ct values above 30, with an overall detection frequency of 71.8% (28/39) (Figure 6B) when compared with the 92.0% (46/50) observed for those with Ct ≤ 30. Advanced modeling corroborated a positive correlation between higher Ct values (low pathogen loads) and detection failure (Figure 7) and consolidated the conclusion that the combined used of Explify and TELEVIR(+) potentiate pathogen detection through this tNGS approach.

Some limitations of our study can be highlighted essentially due to its retrospective design. Nucleic acids extraction was performed by different methodologies and at different laboratories (i.e., either at the NRLs or at the laboratory performing metagenomics using standard procedures; Supplementary Data; Supplementary Table 1), from clinical samples with different storage records. Whereas, this lack of harmonization hampers the evaluation of the impact of these variables on the tNGS performance, it most likely mirrors real scenarios where this approach will be requested. In fact, due to constraints associated with sample volume availability and the need to perform multiple testing in the laboratory of origin, performing *de novo* extraction is frequently unfeasible. Nonetheless, an analysis of possible confounding variables, such as storage time and method of extraction, did not significantly affect tNGS detection rates (Supplementary File). Another important limitation is that the study enrolled clinical samples that had been processed by INSA NRLs for a specific PCR/qPCR diagnosis (as requested by clinicians). As such, due to ethical constraints, the evaluation of the performance of the adopted tNGS method had to strictly focus on identifying microorganisms previously diagnosed by such molecular assays. A full assessment of tNGS will require a prospective comparative study, involving broader and unbiased pathogen search, in order to estimate other performance indicators, such as false positives. Indeed, our evaluation of tNGS performance was limited to the calculation of detection rates and inspection of false negatives, hampering the assessment of whether tNGS could bring additional added value by detecting other pathogens that were not screened through primary PCR diagnosis. For example, for the 10 samples with multiple PCR positive hits (*n* = 25), tNGS could detect 21 hits. Finally, it should be recognized that our analyses on fungi and parasites were not as deep as for bacteria and viruses, since our extended TELEVIR(+) pipeline does not yet cover automatic classification and mapping of these taxa. Despite technical barriers regarding genomic complexity and database completeness, work is ongoing to streamline the bioinformatic analysis of these pathogens.

In summary, the present study demonstrates the tNGS potential to advance personalized medicine by enhancing diagnostic capabilities, which can positively impact the selection of targeted therapies, and ultimately improve patient outcomes. However, this approach still poses several significant challenges, which stem from the complexity of the technology and data interpretation, the need for specialized expertise, the high turnaround time and various logistical and regulatory considerations. Moreover, a close collaboration and communication amongst clinicians and the NRLs are critical, not only to exchange useful clinical/epidemiological information that can guide the laboratory investigation, but also to truly assess the actual impact of the mNGS results on the therapeutics and disease progression. The benefits of clinical metagenomics are expected to increase as some improvements are achieved toward the reduction of turnaround time, workload and costs. Overall, this study allowed the validation and establishment of a pioneering approach for laboratory diagnosis at the NRLs in Portugal, contributing to consolidate tNGS as a valuable and robust tool to support clinicians in complex clinical situations requiring novel diagnosis tools.

## Data Availability

The authors declare that all data supporting the findings of this study are available within the paper and its Supplementary File. Due to ethical considerations (see Ethics Statement), the complete raw read dataset “containing human sequence data” cannot be publicly shared. Instead, reads corresponding to the pathogen(s) primarily detected by specific tests in each sample, and which were used as the basis for determining detection rates throughout the study were released. For each sample, reads were submitted per panel (RPIP or UPIP) and correspond to quality-processed reads mapped to representative genome sequences of the pathogens confirmed by PCR (“PCR positive hits”), being available in the European Nucleotide Archive (ENA) under the BioProject accession numbers PRJEB91208 (accession numbers per sample are detailed in Supplementary Data).
