# Supplementary material for: Probe-based metagenomic pathogen detection: advancing laboratory capacity for complex diagnosis
Source: Front Microbiol. 2025 Oct 14;16:1656831. doi: 10.3389/fmicb.2025.1656831 (PMC12558915; doi:10.3389/fmicb.2025.1656831)
Supplement: Supplementary file 2 [file Data_Sheet_2.pdf]

## Supplementary File

Here we provide additional information detailing the bioinformatics methods and parameters used in the study, TELEVIR's classifiers outputs and performance, and statistical analyses of the impact of confounding variables on the results.

**Contents:**


1. Bioinformatics methods
   1.1. Workflows: Software and versions
   1.2. Other Resources
2. TELEVIR(+) output analysis
   2.1. Classifier output analysis
   2.2. Classifier performance
3. Statistical analyses of confounding variables
   3.1. Students T-test
   3.2. Chi square tests


## 1. Bioinformatics methods

### 1.1. Workflows: Software and versions

Parameters for deployment of bacterial and viral metagenomics for two workflows with different read classification software.

| Module | Software | Value Bacteria | Value Viruses |
|---|---|---|---|
| QC | Trimmomatic v.39[1] | SLIDINGWINDOW:5:20 LEADING:3 TRAILING:3MINLEN:35 TOPHRED33 | SLIDINGWINDOW:5:20 LEADING:3 TRAILING:3 MINLEN:35 TOPHRED33 |
| Extra QC | prinseq++ v1.2.4[2] | --lc_dust 0.7 --lc_entropy 0.5 | --lc_dust 0.7 --lc_entropy 0.5 |
| Pathogen enrichment | kraken2 v2.1.2[3] | --confidence 0.5 --db bacteria --quick | --confidence 0.5 --db viral --quick |
| Host depletion | Bwa v.7.17[4] | --db /televir/mngs_benchmark/ref_db/bwa/hg38/hg38.fa -M | --db /televir/mngs_benchmark/ref_db/bwa/hg38/hg38.fa -M |
| Assembly | SPAdes V3.11.1[5] | -k 31 --phred-offset 33 --only-assembler | -k 31 --phred-offset 33 --only-assembler |
| Contig Classification | kraken2 v2.1.2 | --confidence 0.5 --db bacteria --quick | - |

| | | | |
|---|---|---|---|
| Contig Classification | BLAST v2.12.2[6] | - | -max_target_seqs 5 -evalue 1e-5 --db refseq_viral_genome |
| Read Classification | Centrifuge V1.0 .4_beta[7] | --min-hitlen 22 -k 3 --db bacteria_index | --min-hitlen 22 -k 3 --db viral_index |
| Read Classification | kraken2 v2.1.2 | --quick –confidence 0.5 --db bacteria | --quick –confidence 0.5 --db viral |
| Request Mapping | Snippy v3.2[8] | --mapqual 20 --mincov 2 --minfrac 0.51 | --mapqual 20 --mincov 2 --minfrac 0.51 |

### 1.2. Other Resources

For depletion, samples were depleted of human reads by mapping against the human genome reference assembly hg38 (BioProject PRJNA31257).

The Kraken2 index of the Refseq viral subset was downloaded from https://benlangmead.github.io/aws-indexes/k2, last updated December 06, 2023.

The Kraken2 index of the Refseq prokaryote subset was downloaded from https://benlangmead.github.io/aws-indexes/k2, last updated January 12, 2024.

The NCBI Refseq nucleotide database viral [9] and prokaryote subsets were used for classification by Centrifuge (downloaded December 06 2023 and January 12 2024).

## 2. TELEVIR(+) output analysis

TELEVIR(+) results were pooled and analysed with respect to individual workflows, as well as pooled by sample and detected organism. Relation to PCR and Explify classifications required matching output descriptors.

### 2.1. Classifier output analysis

Classifier software for both Centrifuge and Kraken2 return a number of hits by taxid for each sample. Classifier report hits were sorted by number of hits to determine rank, as performed by TELEVIR automatically.

True positives were determined by matching classifier report hits to descriptions of pathogens verified to be present in each sample by PCR: *i*) descriptions were first extracted from NCBI for the TAXIDs in the reports; *ii*) original pathogen descriptions were processed as follows:

- Descriptions containing "EBV" were labeled as "human herpesvirus 4".
- Descriptions containing "polyomavirus" were labeled as "polyomavirus".
- Descriptions containing "Metapneumovírus" were labeled as "human metapneumovirus".
- Descriptions containing "SARS-CoV-2" were labeled as "Severe acute respiratory syndrome coronavirus".
- Descriptions containing "Varicella" were labeled as "human herpesvirus 3".

- Descriptions containing "Cytomegalovirus" were labeled as "human herpesvirus 5".
- Descriptions containing "Rinovírus" or "Rinovíru" were labeled as "rhinovirus".
- Descriptions containing "Epstein-Barr virus (EBV)" were labeled as "human herpesvirus 4".
- Descriptions containing "Herpes simplex virus 1" were labeled as "human herpesvirus 1".
- Descriptions containing "RSV" were labeled as "respiratory syncytial virus".
- If a description did not contain any of these keywords, it was left as is.

iii) Finally, NCBI descriptions were matched to the original description names using a reciprocal case-insensitive string membership test.

## 2.2. Classifier performance and TELEVIR(+) rank distribution

Although Kraken2 classified 59.77% of Explify identified pathogens, and Centrifuge classified 78.16%, they appeared complementary in their classifications, as there was a considerable overlap (37.93%) between the classifications obtained from Kraken2 and Centrifuge analysis. Both classifiers were then compared in terms of the ranking attributed to pathogens by read count, as reported by each. Across overlapping classifications, we find that Kraken2 outperforms Centrifuge, which ranks pathogens 1.6 times higher (r2 = 0.81). Overall, Centrifuge achieves a marginally better performance, with over 62.0% of pathogens ranking in the 20 first pathogens compared to 48.3% in the case of Kraken2.

The evaluation of the position ranks of ‘PCR-positive hits’ in the sorted TELEVIR(+) classification report, obtained by combining the results of the two classifiers, Centrifuge and Kraken2, show that the vast majority of hits (85,2%) fall below the defined threshold (top 20) for automatic mapping (Supplementary Figure 1), in line with benchmarking results obtained during pipeline development[10].

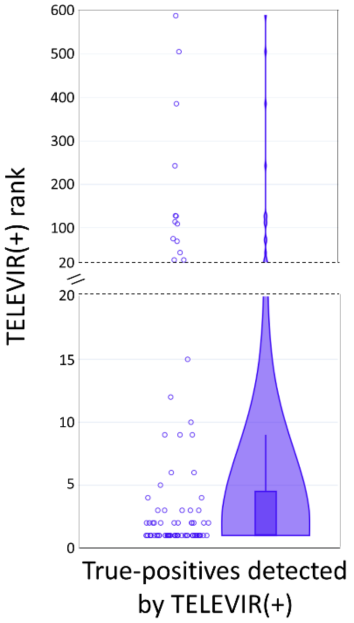


**Supplementary Figure 1**. Violin, scatter and box plots showing the dispersion position ranks of ‘PCR-positive hits’ in the sorted TELEVIR(+) classification report, obtained by combining the results of the two classifiers, Centrifuge and Kraken2.

## 3. Statistical analyses of confounding variables

In this section, the impact of several key variables on the overall pathogen detection rates is evaluated. Given the study design, two primary factors are the extraction methods used and the sample storage duration (from initial collection to tNGS processing). Additionally, since the study encompassed diverse classes of human pathogens, assessing whether Gram-staining classification and genome type (DNA vs. RNA) influenced detection outcomes was desirable, considering their potential role as confounding factors.

### 3.1. Sample storage duration *versus* pathogens' detection

To evaluate the impact of the sample storage duration on pathogens' detection outcomes, a Student's t-test (two-tailed, unpaired) was performed. The null hypothesis ($H_0$) stated that there is no significant difference in the mean storage durations (in days) between the "detected" and "non-detected" pathogen groups. The analysis indicated that extended storage periods did not have a negative effect on the detection results (p=0.543856).

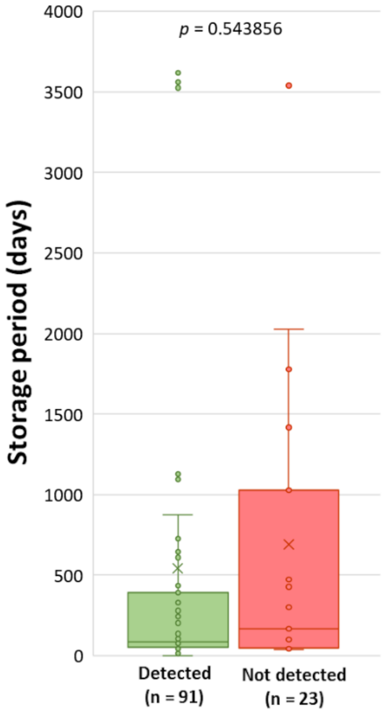


**Supplementary Figure 2.** The graph shows the detection result of the combined approach [Explify and INSaFLU-TELEVIR(+)], in which in green are represented the detected pathogens (n=91) and in red the ones that were not detected (n=23), plotted according to the number of days the respective sample/nucleic acids were stored (from sampling to tNGS). Each box spans the interquartile range (IQR), from the 25th to 75th percentile and the horizontal line within each box indicates the median Ct value of the respective group.

### 3.2. Extraction methods' *versus* pathogens' detection

To evaluate if pathogen detection outcome was influenced by the extraction methods used in this study [the EMAG® (BioMerieux) and the Quick-DNA/RNA

MiniPrep Plus kit (Zymo Research)], a Chi-square ($\chi^2$) test was performed and the null hypothesis could not be rejected ($H_0$: there is no association between the result outcome and the extraction method; $\chi^2(1, N=114) = 1,0878$, p=0.296944).

### 3.3. Bacterial Gram-staining classification *versus* pathogens' detection

As cell wall structure of bacteria may affect the efficiency of some experimental procedures, a Chi-square ($\chi^2$) test was performed to assess if pathogens' detection performed in this study were affected. Again, the null hypothesis could not be rejected ($H_0$: there is no association between the result outcome and the bacterial Gram-staining classification; $\chi^2(1, N=35) = 0,0307$, p=0,860816).

### 3.4. Viral genomic molecule type *versus* pathogens' detection

Viral genome type (DNA vs. RNA) can affect detection due to differences in stability and processing. RNA is more prone to degradation and requires reverse transcription, adding variability. DNA is more stable and easier to process, which may lead to higher detection rates. Therefore, a Chi-square test was used to evaluate whether detection rates differed significantly between DNA and RNA viruses, and no association was found ($H_0$: there is no association between the result outcome and the viral genomic molecule type; $\chi^2(1, N=68) = 0,3185$, p=0,572509).

## REFERENCES


1. Bolger, A. M., Lohse, M. & Usadel, B. Trimmomatic: a flexible trimmer for Illumina sequence data. *Bioinformatics* **30**, 2114–2120 (2014).
2. Cantu, V. A., Sadural, J. & Edwards, R. PRINSEQ++, a multi-threaded tool for fast and efficient quality control and preprocessing of sequencing datasets. at https://doi.org/10.7287/peerj.preprints.27553v1 (2019).
3. Wood, D. E., Lu, J. & Langmead, B. Improved metagenomic analysis with Kraken 2. *Genome Biol.* **20**, 257 (2019).
4. Li, H. Aligning sequence reads, clone sequences and assembly contigs with BWA-MEM. (2013).
5. Prjibelski, A., Antipov, D., Meleshko, D., Lapidus, A. & Korobeynikov, A. Using SPAdes De Novo Assembler. *Curr. Protoc. Bioinforma.* **70**, (2020).
6. Camacho, C. *et al.* BLAST+: architecture and applications. *BMC Bioinformatics* **10**, 421 (2009).
7. Kim, D., Song, L., Breitwieser, F. P. & Salzberg, S. L. Centrifuge: rapid and sensitive classification of metagenomic sequences. *Genome Res.* **26**, 1721–1729 (2016).
8. Torsten Seemann. Snippy: fast bacterial variant calling from NGS reads. https://github.com/tseemann/snippy (2015).
9. Brister, J. R., Ako-adjei, D., Bao, Y. & Blinkova, O. NCBI Viral Genomes Resource. *Nucleic Acids Res.* **43**, D571–D577 (2015).
10. Santos, J. D. *et al.* INSaFLU-TELEVIR: an open web-based bioinformatics suite for viral metagenomic detection and routine genomic surveillance. *Genome Med.* **16**, 61 (2024).
