# Supplementary material for: Probe-based metagenomic pathogen detection: advancing laboratory capacity for complex diagnosis
Source: Front Microbiol. 2025 Oct 14;16:1656831. doi: 10.3389/fmicb.2025.1656831 (PMC12558915; doi:10.3389/fmicb.2025.1656831)
Supplement: Supplementary file 3 [file Table_1.docx]

**Supplementary Table 1**. Sample preparation procedures carried out according to sample nature/matrix prior to DNA and RNA extraction.

| **Sample type** | **Sample preparation** |
| --- | --- |
| Biopsy (mammalian tissue) | · Submerge an appropriate amount of fresh or frozen sample into 350 µL of DNA/RNA Shield™ (1X).  · Homogenise samples by bead beat them with ZR Bashing Bead Lysis Tubes, for 45 seconds at 6,5 m/s, twice, and keeping them in ice for 2 min in between beating cycles.  · For each 350 µL, add 17.5 µL of Proteinase K and 35 µL of PK Digestion Buffer.  · Incubate at 55 ºC, overnight.  · Homogenise samples, once again, by bead beat them with ZR Bashing Bead Lysis Tubes, for 45 seconds at 6.5 m/s, twice, and keeping them in ice for 2 min in between beating cycles.  · Centrifuge and transfer ~250 µL supernatant to a new tube.  · Add 250 µL (1:1) DNA/RNA Lysis Buffer and mix well.  · Continue to purification, page 10, of the Quick-DNA/RNA™ Miniprep Plus Kit instructions handbook *^a^*. |
| Whole blood, serum, plasma | · Add 250 µL of DNA/RNA Shield™ (2X) to 250 µL of sample.  · Add 10 µL Proteinase K and mix well.  · Incubate at room temperature (20-30 °C) for 30 minutes.  · Add 500 µL (1:1) of isopropanol and mix well.  · Transfer sample into a Spin-Away™ Filter (yellow) in a Collection Tube and centrifuge.  · Discard the flow-through and transfer the filter into a new nuclease-free tube.  · Add 200 µL (1:1) DNA/RNA Lysis Buffer directly to filter matrix, let sit for 5 minutes then centrifuge.  · Proceed to purification, page 10, of the Quick-DNA/RNA™ Miniprep Plus Kit instructions handbook *^a^*. |
| Cerebrospinal fluid (CSF), respiratory samples and all swabs | · Add 250 µL of DNA/RNA Shield™ (2X) to 250 µL of sample.  · Add 25 µL Proteinase K and 50 µL PK Digestion Buffer.  · Mix well and incubate at room temperature (20-30 °C) for ≥30 minutes.  · Vortex samples briefly and centrifuge at max speed for 2 minutes.  · Transfer ~500 µL of the cleared supernatant to a nuclease-free tube.  · Add 500 µL (1:1) DNA/RNA Lysis Buffer directly to filter matrix, let sit for 5 minutes then centrifuge.  · Proceed to purification, page 10, of the Quick-DNA/RNA™ Miniprep Plus Kit instructions handbook *^a^*. |

*^a^* Quick-DNA/RNA™ Miniprep Plus Kit instructions handbook at <https://files.zymoresearch.com/protocols/_d7003t_d7003_quick-dna-rna_miniprep_plus_kit.pdf>
